# Tetraquark and two-meson states at large $N_{\mathrm {c}}$

**DOI:** 10.1140/epjc/s10052-017-5437-x

**Published:** 2017-12-13

**Authors:** Wolfgang Lucha, Dmitri Melikhov, Hagop Sazdjian

**Affiliations:** 1 Institute for High Energy Physics, Austrian Academy of Sciences, Nikolsdorfergasse 18, 1050 Vienna, Austria; 2 D. V. Skobeltsyn Institute of Nuclear Physics, M. V. Lomonosov Moscow State University, 119991 Moscow, Russia; 3 Faculty of Physics, University of Vienna, Boltzmanngasse 5, 1090 Vienna, Austria; 4 IPNO, Université Paris-Sud, CNRS-IN2P3, Université Paris-Saclay, 91405 Orsay, France

## Abstract

Considering four-point correlation functions of color-singlet quark bilinears, we investigate, in the large-$N_{\mathrm {c}}$ limit of QCD, the subleading diagrams that involve, in the *s*-channel of meson–meson scattering amplitudes, two-quark–two-antiquark intermediate states. The latter contribute, together with gluon exchanges, to the formation, at the hadronic level, of two-meson and tetraquark intermediate states. It is shown that the two-meson contributions, which are predictable, in general, from leading-order $N_{\mathrm {c}}$-behaviors, consistently satisfy the constraints resulting from the $1/N_{\mathrm {c}}$ expansion procedure and thus provide a firm basis for the extraction of tetraquark properties from $N_{\mathrm {c}}$-subleading diagrams. We find that, in general, tetraquarks, if they exist in compact form, should have narrow decay widths, of the order of $N_{\mathrm {c}}^{-2}$. For the particular case of exotic tetraquarks, involving four different quark flavors, two different types of tetraquark are needed, each having a preferred decay channel, to satisfy the consistency constraints.

## Introduction

The existence of tetraquarks as tightly bound states of QCD [1, 2], also called compact tetraquarks, is still a matter of theoretical debate. The problem is related to the issue as to whether the interquark confining forces may produce bound states made essentially of a pair of quarks and a pair of antiquarks, just as they produce ordinary meson states made of a quark and an antiquark. A questioning may arise from the fact that interpolating color-neutral four-quark local operators that would create tetraquark states can be decomposed by Fierz rearrangements into combinations of products of color-neutral quark bilinears [3], which are rather suggestive of the creation of pairs of free ordinary meson states. In the absence of an exact resolution of the four-body problem in the presence of confining forces, simplified models, based on the diquark and antidiquark associations, have been proposed, in which the existence of attractive forces, favoring the formation of tetraquark bound states, is more transparent [4–8]. Lattice calculations do not yet bring firm conclusions, the results often depending on the flavor of the heavy quarks that are considered [9–13].

On the other hand, it is generally admitted that in ’t Hooft’s large-$N_{\mathrm {c}}^{}$ limit of QCD [14], with the coupling constant *g* scaling as $N_{\mathrm {c}}^{-1/2}$ and with the quark fields belonging to the fundamental representation of the color gauge group ${\mathrm {SU}}(N_{\mathrm {c}}^{})$, the theory catches the main properties of confinement, while being liberated from secondary screening phenomena, such as quark pair creation or inelasticity effects. Applying this approach to color-neutral quark bilinear operators, Witten has shown that in this limit the related QCD correlation functions are saturated by noninteracting meson states [15]. Generalizing the application to color-neutral quark quadrilinear operators, Coleman then showed that the correlation functions of the latter are dominated, in the large-$N_{\mathrm {c}}^{}$ limit, by free ordinary meson states [16].

For a long time, the latter result has been considered as an indication for the nonexistence of tetraquarks as bound states surviving the large-$N_{\mathrm {c}}^{}$ limit. Recently, however, Weinberg has reexamined the question by noticing the fact that the subleading nature of the interaction part of the quark quadrilinear operators is not a proof of the nonexistence of tetraquarks, but rather might be a constraint on their decay widths. Considering a class of candidate operators, he showed that if tetraquarks exist in the large-$N_{\mathrm {c}}^{}$ limit as bound states with finite masses, then they should have narrow widths, of the order of $N_{\mathrm {c}}^{-1}$, like those of the ordinary mesons, and would thus be observable [17]. In complement to the latter result, Knecht and Peris have stressed that depending on their flavor content, tetraquarks might even be narrower in some cases, having widths of the order of $N_{\mathrm {c}}^{-2}$ [18]. Cohen and Lebed, studying the analyticity properties of meson–meson scattering amplitudes, have reported that in the case of exotic tetraquarks the decay widths should, in general, be of the order of $N_{\mathrm {c}}^{-2}$ or less [19]. Reference [20] reported the possibility of smaller widths of the order of $N_\mathrm {c}^{-3}$. The $N_\mathrm {c}^{}$-analysis of meson–meson scattering amplitudes, in connection with lattice calculations, has also been presented in [21].

The aim of the present paper is to investigate in a systematic way the $N_{\mathrm {c}}^{}$-subleading diagrams where tetraquark candidates may occur. They are characterized by the presence of two-quark–two-antiquark intermediate states^1^ in the *s*-channel of meson–meson scattering amplitudes. Here, however, an additional complication arises with respect to the usual cases of $N_{\mathrm {c}}^{}$-leading diagrams: four-quark intermediate states also signal the presence of two interacting meson states at the hadronic level. Since the $N_{\mathrm {c}}^{}$-behavior of three-meson and four-meson vertices can be determined, in general, from simpler diagrams, the subleading two-meson contributions are then completely predicted and thus should satisfy consistency checks within the above analysis. The latter is a crucial test for the validity of the $1/N_{\mathrm {c}}^{}$-expansion method in QCD. It is once that these contributions are evaluated and tested that one may safely extract the properties of tetraquarks from the $N_{\mathrm {c}}$-subleading diagrams.

Concerning the four-quark intermediate states, their presence should be determined with the aid of the Landau equations [22, 23], which provide unambiguous criteria for their existence. It is also understood that each QCD diagram with four-quark intermediate states is accompanied by similar diagrams with insertions of any number of gluon lines neither changing its topology, nor its $N_{\mathrm {c}}$-behavior; it is the infinite sum of such diagrams that produces the hadronic-state singularities, as tetraquark poles or two-meson cuts.

An important ingredient in the present approach is provided by the consideration, for a given set of quark flavors, of all possible meson–meson scattering channels which may produce four-quark singularities; in this way, one obtains the maximum number of constraints on the properties of the tetraquark candidates, which often are not apparent within a single channel. We also emphasize that no hypothesis is done about the internal structure of the tetraquark states with respect to the possible combinations of quark fields. In some cases, the existing constraints are strong enough to suggest the most favorable structures.

Our main results can be summarized as follows. First, the two-meson contributions, which emerge through effective meson one-loop diagrams, satisfy all the consistency checks coming from the $N_{\mathrm {c}}$-behaviors of three- and four-meson vertices. This confirms the validity of the perturbative $1/N_{\mathrm {c}}^{}$ expansion to the next-to-leading-order diagrams. Second, tetraquarks, if they exist, should have, in general, narrow decay widths, of the order of $N_{\mathrm {c}}^{-2}$, much smaller than those of ordinary mesons, which are of the order of $N_{\mathrm {c}}^{-1}$. Third, in the case of exotic sectors, involving four different quark flavors, two different tetraquarks, each having a preferred decay channel, are needed to fulfill the consistency conditions. The internal structure of these tetraquarks, expressed as a quark quadrilinear, would be of the form of a product of two color-singlet bilinears. Part of the results above has been presented in [24].

The generality of the tetraquark width estimate of being of the order of $N_{\mathrm {c}}^{-2}$, obtained in the present paper, in contradistinction with the weaker estimate of Ref. [17], $O(N_{\mathrm {c}}^{-1})$, stems from the fact that color-planar diagrams, with one external quark loop and without internal quark loops, do not have *s*-channel four-quark singularities. This eliminates the potential presence of tetraquark intermediate states in such types of diagram and consequently reduces the magnitude of the tetraquark–two-meson transition amplitudes. This feature has been overlooked in Ref. [17].

We concentrate, in the following (Sects. 2 and 3), on the cases of exotic and cryptoexotic channels, corresponding to four and three different quark flavors, respectively. The case of two flavors can be treated in a similar way to three flavors and is briefly sketched. Details of the calculations related to the role of the Landau equations are presented in the appendix.

For recent reviews on the experimental properties of tetraquark candidates and their theoretical interpretations, we refer the reader to Refs. [25–35].

## Exotic channels

Our analysis is based on the study of four-point correlation functions of color-singlet meson sources or currents *j* of the type $\langle jjj^{\dagger }j^{\dagger }\rangle$, where the *j* are specified by their quark flavor content. We define

2.1 $$\begin{aligned} j_{ab}^{}=\overline{q}_a^{}\hat{O}q_b^{}, \end{aligned}$$

where *a* and *b* are flavor indices and $\hat{O}$ is a combination of Dirac matrices. We do not focus in this work on the detailed spin and parity structure of mesons and tetraquarks, which does not play a fundamental role in the subsequent analysis, and hence we shall omit all Lorentz structures. An ordinary meson, having the flavor content of antiquark *a* and quark *b*, will be designated by $M_{ab}^{}$; its coupling to the current $j_{ab}^{}$ is designated by $f_{M_{ab}^{}}$:

2.2 $$\begin{aligned} \langle 0|j_{ab}^{}|M_{ab}^{}\rangle = f_{M_{ab}^{}}^{} =O(N_{\mathrm {c}}^{1/2}), \end{aligned}$$

where we have also indicated its large-$N_{\mathrm {c}}^{}$ behavior [15].

We concentrate in this section on fully exotic channels, in which four different quark flavors are present, designated by the labels *a*, *b*, *c*, *d*. We consider the four-point correlation functions, classified in the *s*-channels as “direct” I and II and “recombination”, respectively,

2.3 $$\begin{aligned}&\varGamma _{\mathrm {I}}^{\mathrm {dir}}=\langle j_{ab}^{}j_{cd}^{} j_{cd}^{\dagger }j_{ab}^{\dagger }\rangle ,\quad \varGamma _{\mathrm {II}}^{\mathrm {dir}}=\langle j_{ad}^{}j_{cb}^{} j_{cb}^{\dagger }j_{ad}^{\dagger }\rangle ,\nonumber \\&\varGamma ^{\mathrm {rec}}=\langle j_{ab}^{}j_{cd}^{}j_{cb}^{\dagger } j_{ad}^{\dagger }\rangle ,\quad a\ne b\ne c\ne d. \end{aligned}$$

**Fig. 1:**
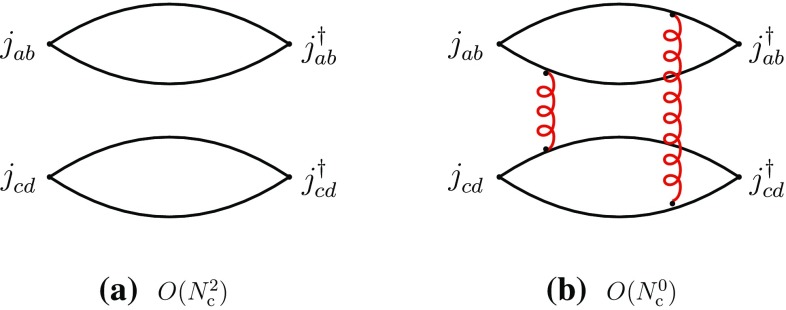
Leading- and subleading-order diagrams of the “direct” channel I of Eq. (2.3). **a** Disconnected part; **b** connected part (a sample diagram). Full lines represent quarks, curly lines gluons. Similar diagrams exist for the “direct” channel II

We first consider the direct channels. The corresponding correlation functions have a disconnected part representing the propagation of two free mesons $M_{ab}^{}$ and $M_{cd}^{}$, or $M_{ad}^{}$ and $M_{cb}^{}$, and producing a global dependence of leading order $N_{\mathrm {c}}^2$, and a connected part, containing at least two-gluon exchanges between the disconnected pieces, and having a leading-order behavior of $N_{\mathrm {c}}^0$ (Fig. 1).

It is evident that only the connected part of the above correlation functions can have any information as regards the meson–meson interaction. To isolate the latter, one extracts from the connected part of the correlation function the related scattering amplitude by factorizing the external four meson propagators together with the related couplings (2.2). These diagrams have four-quark singularities in the *s*-channel (cf. the appendix) and hence are saturated, at $N_{\mathrm {c}}$-leading order, by intermediate states composed of two interacting mesons and of tetraquarks, designated by *T*. One obtains the following leading-order behaviors for the two-meson scattering amplitudes and the transition amplitudes through two-meson and tetraquark intermediate states:

2.4 $$\begin{aligned}&A(M_{ab}^{}M_{cd}^{}\rightarrow M_{ab}^{}M_{cd}^{}) \sim A(M_{ad}^{}M_{cb}^{}\rightarrow M_{ad}^{}M_{cb}^{})\nonumber \\&\quad = O(N_{\mathrm {c}}^{-2}), \end{aligned}$$

2.5 $$\begin{aligned}&A(M_{ab}^{}M_{cd}^{}\rightarrow MM\rightarrow M_{ab}^{}M_{cd}^{})\nonumber \\&\quad \sim A(M_{ad}^{}M_{cb}^{} \rightarrow MM\rightarrow M_{ad}^{}M_{cb}^{}) = O(N_{\mathrm {c}}^{-2}), \end{aligned}$$

2.6 $$\begin{aligned}&A(M_{ab}^{}M_{cd}^{}\rightarrow T\rightarrow M_{ab}^{}M_{cd}^{}) \nonumber \\&\quad \sim A(M_{ad}^{}M_{cb}^{} \rightarrow T\rightarrow M_{ad}^{}M_{cb}^{}) = O(N_{\mathrm {c}}^{-2}). \end{aligned}$$

**Fig. 2:**
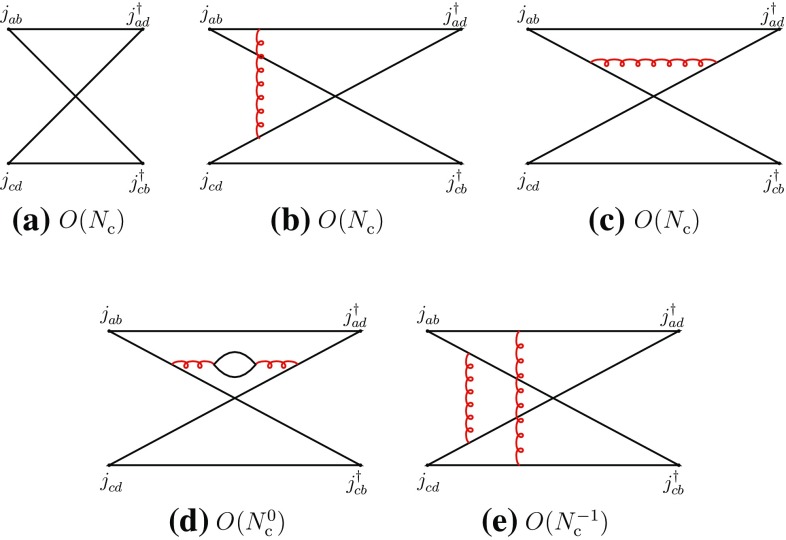
Leading- and typical subleading-order diagrams of the “recombination” channel of Eq. (2.3)

Next, we consider the recombination channel of (2.3). Here, there are no disconnected diagrams and the leading-order behavior is $O(N_{\mathrm {c}})$ (Fig. 2).

Using the Landau equations, one checks that Fig. 2a–d do not have *s*-channel singularities [cf. Appendix]. Their singularities arise in the *u*- and *t*-channels. (However, Fig. 2a–c do not have four-quark cuts in any channel.) Figure 2e is the first diagram where four-quark singularities appear in the *s*-channel and hence it may contribute to meson–meson scattering with two-meson and tetraquark intermediate states.

**Fig. 3:**
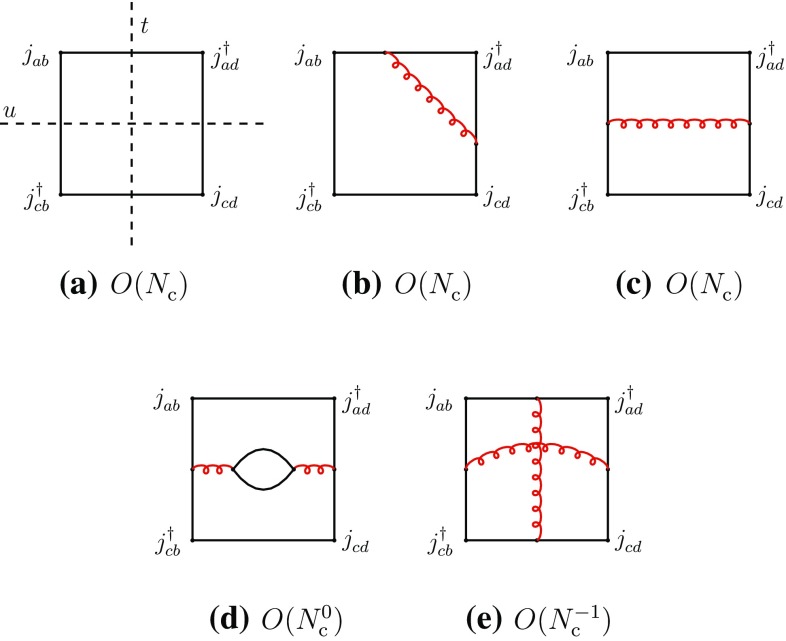
The diagrams of Fig. 2 in unfolded form

The previous properties can also be understood in terms of the topological properties of the diagrams in color-space. Figure 2a–d are color-planar, while Fig. 2e is color-nonplanar. This can be more easily seen by unfolding the diagrams to make the color flow apparent (Fig. 3). The unfolded plane corresponds now to the (*u*, *t*) plane. The manifest singularities in the color-planar diagrams correspond to those of the *u*- and *t*-channels, obtained with vertical and horizontal cuts, respectively. *s*-channel singularities can be searched for by cutting the box-diagrams with oblique and curved lines passing through the four quark propagators. However, when the diagram is color-planar, these cuts produce, generally, disconnected singularities concentrated at opposite corners and corresponding to radiative corrections of the external meson propagators and of the current vertices. Therefore no *s*-channel singularities arise here. The latter may arise only when the diagrams are color-nonplanar, because of the specific routing of the momenta. This is precisely the case of Fig. 3e (or its equivalent Fig. 2e). These properties do not depend on the number and configuration of gluon lines, but only on the color-topology of the diagram and could be verified on explicit examples.

Diagrams of the type of Figs. 2d or 3d involve, through the internal quark loop creation, four-quark intermediate states and might participate in the formation of tetraquark states in the *u*-channel. However, since the internal quark loop involves a quark and an antiquark corresponding to the same flavor, the resulting tetraquark would belong to the class of cryptoexotic states; the latter type of tetraquark will be explicitly considered in Sect. 3 and hence *u*- and *t*-channel four-quark singularities will not be analyzed here.

Figure 2a–d also contribute to four-meson vertex-type couplings, which are free of singularities.

One obtains the following behaviors for the two-meson scattering amplitudes and the transition amplitudes through two-meson and tetraquark intermediate states:

2.7 $$\begin{aligned}&A(M_{ab}^{}M_{cd}^{}\rightarrow M_{ad}^{}M_{cb}^{}) \sim A(M_{ad}^{}M_{cb}^{}\rightarrow M_{ab}^{}M_{cd}^{})\nonumber \\&\quad = O(N_{\mathrm {c}}^{-1}), \end{aligned}$$

2.8 $$\begin{aligned}&A(M_{ab}^{}M_{cd}^{}\rightarrow MM\rightarrow M_{ad}^{}M_{cb}^{})\nonumber \\&\quad \sim A(M_{ad}^{}M_{cb}^{}\rightarrow MM\rightarrow M_{ab}^{}M_{cd}^{}) = O(N_{\mathrm {c}}^{-3}), \end{aligned}$$

2.9 $$\begin{aligned}&A(M_{ab}^{}M_{cd}^{}\rightarrow T\rightarrow M_{ad}^{}M_{cb}^{})\nonumber \\&\quad \sim A(M_{ad}^{}M_{cb}^{}\rightarrow T\rightarrow M_{ab}^{}M_{cd}^{}) = O(N_{\mathrm {c}}^{-3}). \end{aligned}$$

**Fig. 4:**
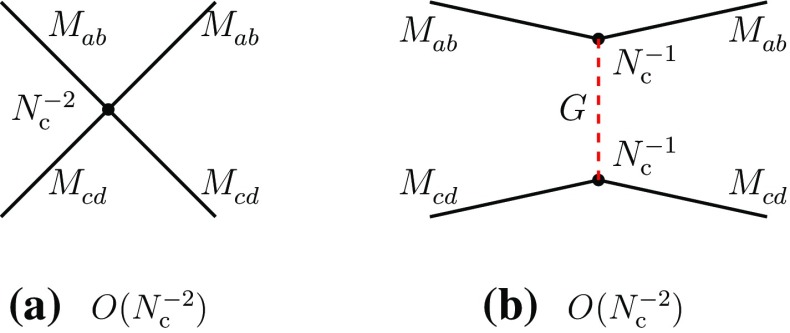
**a** Four-meson vertex in the direct channel I of Eq. (2.3); **b** glueball exchange in the same channel. Similar diagrams also exist in the direct channel II

**Fig. 5:**
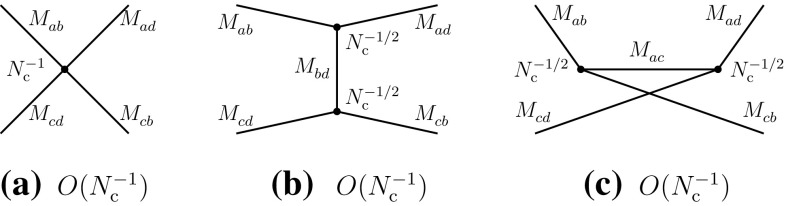
Leading-order meson diagrams in the recombination channel; diagrams of the type of Fig. 2d contribute as meson loop radiative corrections or as a meson–cryptoexotic tetraquark mixing to diagram (c) above

We first analyze Eqs. (2.4) and (2.7) in terms of effective meson vertices. One deduces that the four-meson vertices of the direct type are of order $N_{\mathrm {c}}^{-2}$, while that of the recombination type is of order $N_{\mathrm {c}}^{-1}$ (Figs. 4 and 5):

2.10 $$\begin{aligned}&g(M_{ba}^{}M_{dc}^{}M_{ab}^{}M_{cd}^{}) \sim g(M_{da}^{}M_{bc}^{}M_{ad}^{}M_{cb}^{}) = O(N_{\mathrm {c}}^{-2}),\nonumber \\ \end{aligned}$$

2.11 $$\begin{aligned}&g(M_{da}^{}M_{bc}^{}M_{ab}^{}M_{cd}^{}) = O(N_{\mathrm {c}}^{-1}). \end{aligned}$$

The difference of behavior between the two types of coupling is a consequence of the topological difference between the connected recombination and direct diagrams. The former (Fig. 2a) is planar, the latter (Fig. 1b) is typical of OZI-suppressed diagrams [36–38], made connected by gluon exchanges between two disconnected pieces. This can also be checked in theoretically founded meson effective theories. Since our evaluations do not depend upon masses and momenta, one can consider chiral perturbation theory [39], extended to ${\mathrm {SU}}(4)\times {\mathrm {SU}}(4)$. It can be verified that at the tree level, where four-meson couplings are, in general, of order $N_{\mathrm {c}}^{-1}$, the vertices of the direct channels of (2.3) are absent, thus confirming the results (2.10).

**Fig. 6:**
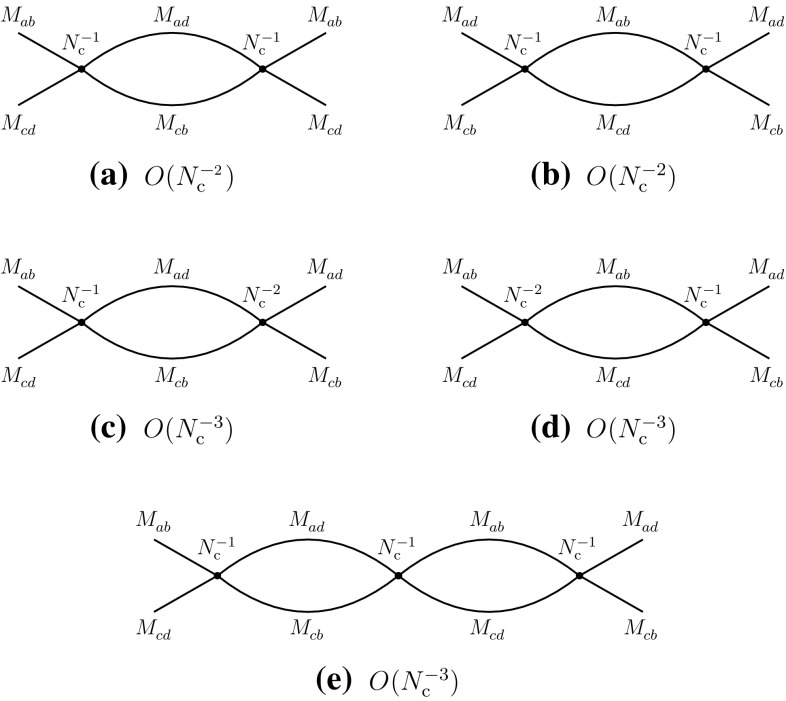
Leading-order contributions of two-meson states to the direct (**a**, **b**) and recombination (**c**–**e**) channels

With the properties of four-meson vertices determined, one can then evaluate the contributions of the *s*-channel two-meson intermediate states in the above processes. The results are summarized in the diagrams of Fig. 6. One observes that they consistently reproduce the behaviors expected from (2.5) and (2.8), corresponding to the diagrams of Figs. 1b and 2e. In particular, going back to Fig. 1b and cutting the diagram with a vertical line between the gluon lines, one finds that the intermediate states are created by the singlet operators that make the mesons $M_{ad}^{}$ and $M_{cb}^{}$, which precisely are the intermediate states of Fig. 6a. A similar check can be done with the other diagrams.

**Fig. 7:**
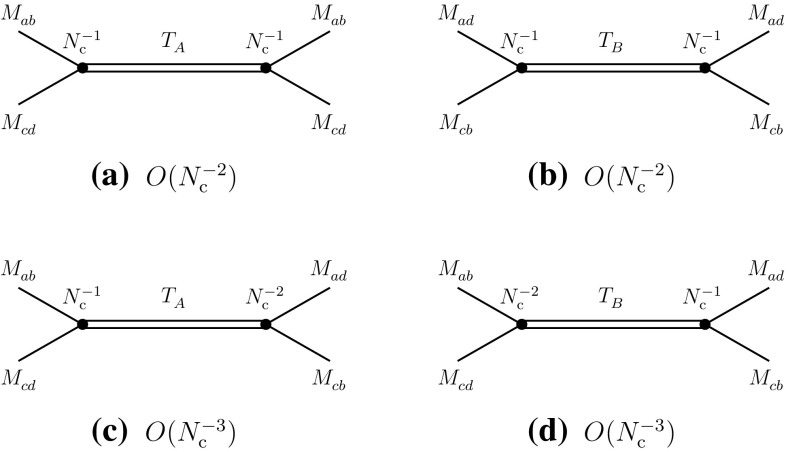
Leading-order contributions of tetraquarks $T_A^{}$ and $T_B^{}$ to the direct (**a**, **b**) and recombination (**c**, **d**) channels

Meson loops generally display ultraviolet divergences. These are essentially absorbed in vertex and propagator renormalizations. The four-meson vertices being, in general, momentum dependent, new types of vertices may emerge, generating with higher-order loops an infinite series of terms in the meson effective Lagrangian. A typical example of that mechanism can be found in chiral perturbation theory [39]. For the purposes of the present approach two remarks are of order. First, as can be observed from the previous examples and diagrams, meson loops have weaker or at most equal dependences on $N_{\mathrm {c}}^{}$ than the generating four-meson vertices. Therefore, vertex renormalizations cannot alter the leading $N_{\mathrm {c}}^{}$-behaviors of existing tree-level vertices. Second, since in our approach we are not displaying the detailed momentum dependence of the vertices, the eventual appearance of new types of vertex does not need the introduction of new tree-level couplings, the existing ones representing generic types.

The possible contributions of tetraquarks can be extracted from Eqs. (2.6) and (2.9). It is evident that a single tetraquark alone cannot satisfy these two equations, unless one runs into contradictions. At least two different tetraquarks, which we denote $T_A^{}$ and $T_B^{}$, are necessary to fulfill the above conditions. The results are summarized as follows and in Fig. 7:

2.12 $$\begin{aligned}&A(T_A^{}\rightarrow M_{ab}^{}M_{cd}^{})\sim O(N_{\mathrm {c}}^{-1}), \nonumber \\&\quad A(T_B^{}\rightarrow M_{ad}^{}M_{cb}^{})\sim O(N_{\mathrm {c}}^{-1}), \end{aligned}$$

2.13 $$\begin{aligned}&A(T_A^{}\rightarrow M_{ad}^{}M_{cb}^{})\sim O(N_{\mathrm {c}}^{-2}), \nonumber \\&\quad A(T_B^{}\rightarrow M_{ab}^{}M_{cd}^{})\sim O(N_{\mathrm {c}}^{-2}). \end{aligned}$$

The decay widths of the tetraquarks are

2.14 $$\begin{aligned} \varGamma (T_A) \sim \varGamma (T_B)=O(N_{\mathrm {c}}^{-2}), \end{aligned}$$

which are smaller than those of the ordinary mesons [$\varGamma =O(N_{\mathrm {c}}^{-1})$] by one power of $N_{\mathrm {c}}^{}$.

The above properties provide us with an indication as regards the internal structure of the tetraquark candidates. Transcribing the four-meson couplings (2.10) and (2.11) (Figs. 4a and 5a) into an effective interaction Lagrangian expressed by means of the corresponding quark color-singlet bilinears, one obtains

2.15 $$\begin{aligned} {\mathcal {L}}_{\mathrm {eff,int}}= & {} -\frac{\lambda _1^{}}{N_c^{}} [(\overline{q}_a^{}q_b^{})(\overline{q}_c^{}q_d^{}) (\overline{q}_d^{}q_a^{})(\overline{q}_b^{}q_c^{})\nonumber \\&+\,(\overline{q}_a^{}q_d^{})(\overline{q}_c^{}q_b^{}) (\overline{q}_b^{}q_a^{})(\overline{q}_d^{}q_c^{})]\nonumber \\&-\,\frac{\lambda _2^{}}{N_c^{2}} [(\overline{q}_a^{}q_b^{})(\overline{q}_c^{}q_d^{}) (\overline{q}_d^{}q_c^{})(\overline{q}_b^{}q_a^{})\nonumber \\&+\,(\overline{q}_a^{}q_d^{})(\overline{q}_c^{}q_b^{}) (\overline{q}_b^{}q_c^{})(\overline{q}_d^{}q_a^{})], \end{aligned}$$

where we have explicitly factored out the $N_\mathrm {c}^{}$-dependence of the coupling constants. One then deduces from Eqs. (2.12) and (2.13) that the tetraquark fields $T_A^{}$ and $T_B^{}$ should have the following structure in terms of the quark color-singlet bilinears:

2.16 $$\begin{aligned} T_A^{} \sim (\overline{q}_a^{}q_d^{})(\overline{q}_c^{}q_b^{}),\quad T_B^{} \sim (\overline{q}_a^{}q_b^{})(\overline{q}_c^{}q_d^{}), \end{aligned}$$

additional mixings between the two, of order $N_{\mathrm {c}}^{-1}$, being still possible.

Manifestly, the above result favors a color singlet-singlet structure of the tetraquarks in the exotic case. It is an open question whether the interquark confining forces may produce bound states with such a structure.

One might also encounter an intermediate situation, where one of the tetraquarks, $T_B^{}$, say, is absent from the spectrum for some dynamical reason. In that case, one tetraquark ($T_A^{}$) would exist and, if the corresponding phase space is favorable, it would be observed through its preferred decay channel, as shown in (2.12).

## Cryptoexotic channels

We next consider cryptoexotic channels, with three different quark flavors, *a*, *b*, *c*, involved within the mesons $M_{ac}^{}$, $M_{cb}^{}$, $M_{ab}^{}$ and $M_{cc}^{}$ [Eqs. (2.1) and (2.2)]. Here also, one may distinguish between direct (I and II) and recombination channels, described by the correlation functions

3.1 $$\begin{aligned}&\varGamma _{\mathrm {I}}^{\mathrm {dir}}=\langle j_{ac}^{}j_{cb}^{} j_{cb}^{\dagger }j_{ac}^{\dagger }\rangle ,\quad \varGamma _{\mathrm {II}}^{\mathrm {dir}}=\langle j_{ab}^{}j_{cc}^{} j_{cc}^{\dagger }j_{ab}^{\dagger }\rangle ,\nonumber \\&\varGamma ^{\mathrm {rec}}=\langle j_{ac}^{}j_{cb}^{}j_{cc}^{\dagger } j_{ab}^{\dagger }\rangle ,\quad a\ne b\ne c. \end{aligned}$$

**Fig. 8:**
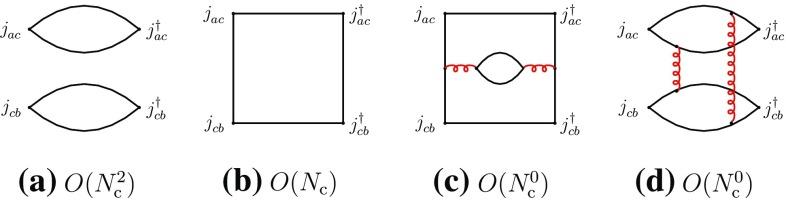
Leading- and typical subleading-order diagrams of the direct channel I of Eq. (3.1)

For the direct channel I, the leading and subleading diagrams are represented in Fig. 8.

Diagram (b) of Fig. 8 represents the leading-order contribution to the meson–meson scattering amplitude. It has only a two-quark singularity in the *s*-channel and therefore represents the contribution of a single-meson intermediate state.

Diagram (c) represents contributions from radiative corrections to the previous diagram. In its utmost left part, one has an intermediate state made of a quark–antiquark pair and a single gluon, which later becomes a four-quark intermediate state and then retrieves back the former situation. In the space of meson states, the first intermediate state contributes to the formation of a single-meson state, which then emits two virtual mesons, or a tetraquark, and reabsorbs them later. Summing the chain of contributions of such types of diagram, one ends up with a radiative correction to the one-meson propagator with a subleading order in $N_{\mathrm {c}}$. This diagram may also describe a mixing between a single-meson state and a tetraquark state, having the same quantum numbers. We shall come back to this question when discussing the mixing problem in the recombination channel.

Diagram (d) represents a direct contribution of two-meson states and/or of a tetraquark state. One obtains the following leading-order behaviors for the two-meson scattering amplitudes and the transition amplitudes through two-meson and tetraquark intermediate states:

3.2 $$\begin{aligned}&A(M_{ac}^{}M_{cb}^{}\rightarrow M_{ac}^{}M_{cb}^{}) = O(N_{\mathrm {c}}^{-1}), \end{aligned}$$

3.3 $$\begin{aligned}&A(M_{ac}^{}M_{cb}^{}\rightarrow MM\rightarrow M_{ac}^{}M_{cb}^{}) = O(N_{\mathrm {c}}^{-2}), \end{aligned}$$

3.4 $$\begin{aligned}&A(M_{ac}^{}M_{cb}^{}\rightarrow T\rightarrow M_{ac}^{}M_{cb}^{}) = O(N_{\mathrm {c}}^{-2}). \end{aligned}$$

**Fig. 9:**
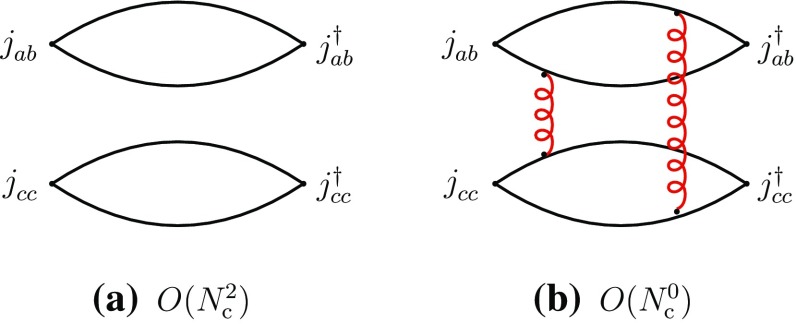
Leading- and subleading-order diagrams of the direct channel II of Eq. (3.1)

For the direct channel II, the structure of the diagrams is similar to that of Fig. 1, represented in Fig. 9, from which one deduces

3.5 $$\begin{aligned}&A(M_{ab}^{}M_{cc}^{}\rightarrow M_{ab}^{}M_{cc}^{}) = O(N_{\mathrm {c}}^{-2}), \end{aligned}$$

3.6 $$\begin{aligned}&A(M_{ab}^{}M_{cc}^{}\rightarrow MM\rightarrow M_{ab}^{}M_{cc}^{}) = O(N_{\mathrm {c}}^{-2}), \end{aligned}$$

3.7 $$\begin{aligned}&A(M_{ab}^{}M_{cc}^{}\rightarrow T\rightarrow M_{ab}^{}M_{cc}^{}) = O(N_{\mathrm {c}}^{-2}). \end{aligned}$$

**Fig. 10:**
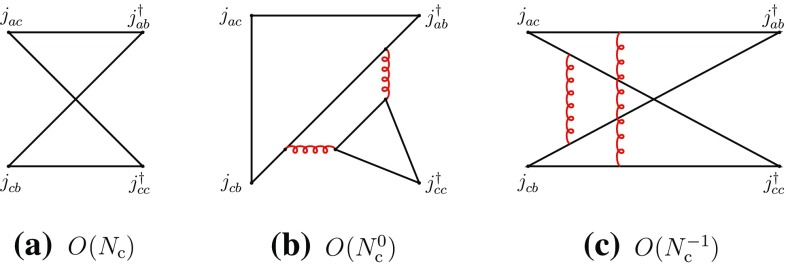
Leading- and typical subleading-order diagrams of the recombination channel of Eq. (3.1)

For the recombination channel of Eq. (3.1), the main leading and subleading diagrams are shown in Fig. 10. (The diagram similar to that of Fig. 2d is not drawn, since it contributes to subleading radiative corrections in the *u*-channel.) Diagram (a) does not have *s*-channel singularities [cf. Figs. 2a and 5a and the appendix], while diagrams (b) and (c) receive contributions from four-quark intermediate states in the *s*-channel (cf. the appendix).

One then deduces the following properties of the meson–meson scattering amplitudes and the transition amplitudes through two-meson and tetraquark intermediate states:

3.8 $$\begin{aligned}&A(M_{ac}^{}M_{cb}^{}\rightarrow M_{ab}^{}M_{cc}^{}) = O(N_{\mathrm {c}}^{-1}), \end{aligned}$$

3.9 $$\begin{aligned}&A(M_{ac}^{}M_{cb}^{}\rightarrow MM\rightarrow M_{ab}^{}M_{cc}^{}) = O(N_{\mathrm {c}}^{-2}), \end{aligned}$$

3.10 $$\begin{aligned}&A(M_{ac}^{}M_{cb}^{}\rightarrow T\rightarrow M_{ab}^{}M_{cc}^{}) = O(N_{\mathrm {c}}^{-2}). \end{aligned}$$

**Fig. 11:**
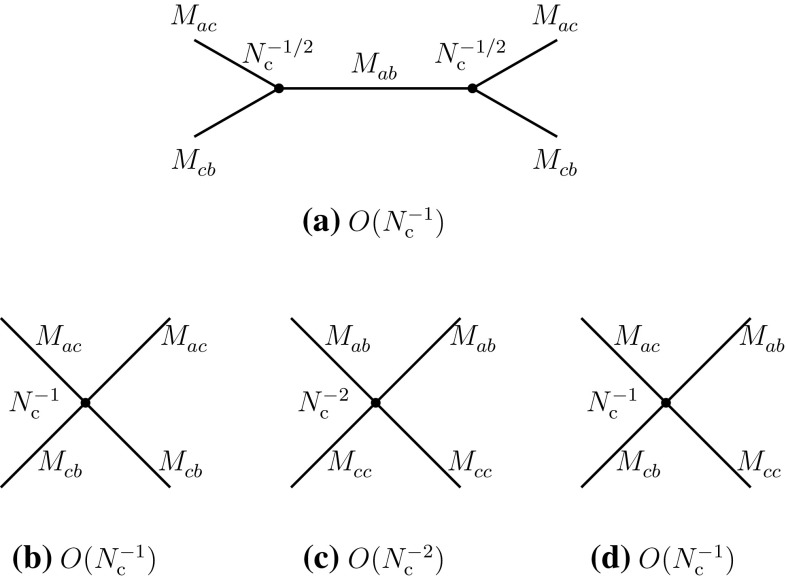
Significant tree-level vertex diagrams with meson propagators in the direct channel I (**a**, **b**), the direct channel II (**c**) and the recombination channel (**d**)

The information obtained about the $N_{\mathrm {c}}^{}$-behaviors of leading and subleading diagrams can now be transcribed into properties of effective meson–meson interactions. These are summarized in Fig. 11.

**Fig. 12:**
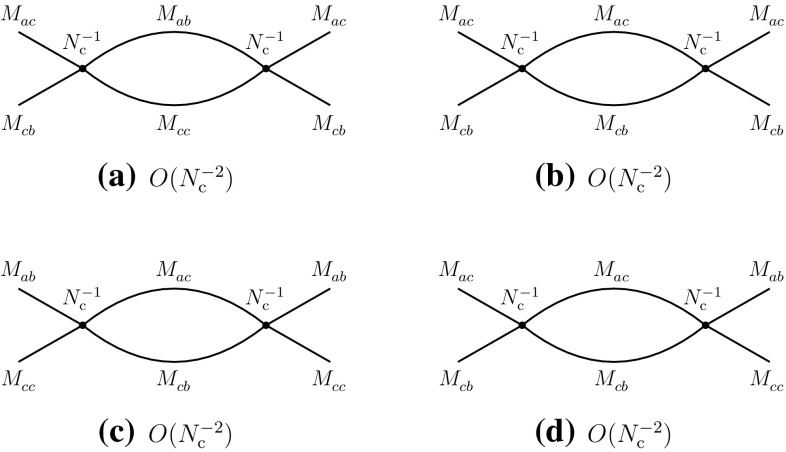
Two-meson intermediate-state contributions to the three channels at $N_{\mathrm {c}}^{}$-leading order: direct channel I (**a**, **b**), direct channel II (**c**) and recombination channel (**d**)

One can then evaluate the contributions of two-meson intermediate states in the scattering amplitudes. The results, for the leading terms, are presented in Fig. 12. They manifestly reproduce the $N_{\mathrm {c}}^{}$-behaviors as expected from Eqs. (3.3), (3.6) and (3.9).

**Fig. 13:**
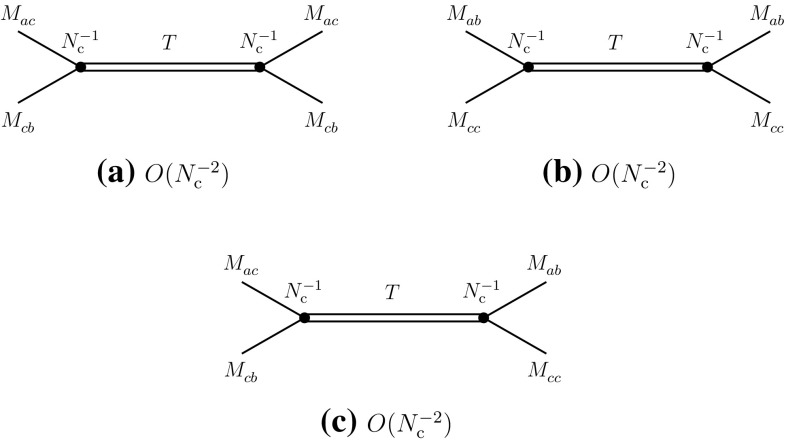
Tetraquark-state contributions to the three channels at $N_{\mathrm {c}}^{}$-leading order: direct channel I (**a**), direct channel II (**b**) and recombination channel (**c**)

The tetraquark contributions can be extracted in the same way as for the exotic channels. Because of the presence of the additional diagram (b) of Fig. 10, they are of the same order in all three channels and hence a single tetraquark *T* can accommodate all the corresponding constraints. The results are summarized in Fig. 13. The decay width of the tetraquark is again of order $N_{\mathrm {c}}^{-2}$:

3.11 $$\begin{aligned} \varGamma (T)=O(N_{\mathrm {c}}^{-2}). \end{aligned}$$

**Fig. 14:**
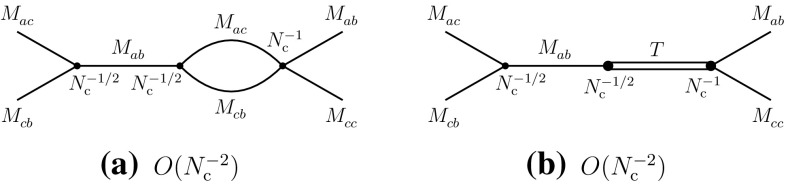
Mixings, in the recombination channel, of a single-meson state with two-meson (**a**) and tetraquark (**b**) states

Actually, diagram (b) of Fig. 10 may also describe a mixing of two-meson states or of a tetraquark state with a single-meson state that appears in the left part of the diagram. The corresponding mixings, which do not change the previous results, are described in Fig. 14.

**Fig. 15:**
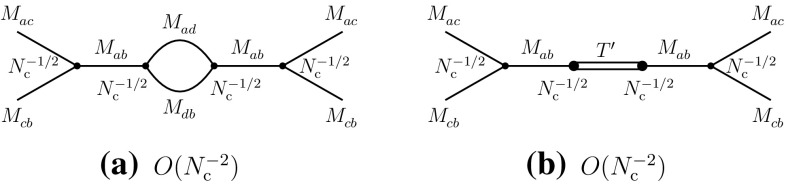
Mixings, in the direct channel I, of a single-meson state with two-meson (**a**) and tetraquark (**b**) states. The flavor of the internal loop quark is designated by *d* and the corresponding tetraquark by $T'$

Meson-tetraquark mixings also exist in the direct channel I, as was previously mentioned, emerging from diagrams of the type of Fig. 8c. Here, since the quark and its antiquark can be created with any flavor, the resulting two-meson and/or tetraquark states may belong to another class of cryptoexotic states. The corresponding mixings are described in Fig. 15. They do not change the leading coupling properties of cryptoexotic tetraquarks to two-meson states.

Cryptoexotic tetraquarks have therefore the possibility of decaying into two mesons either through direct coupling or through mixing with single-meson states. Both types of decay lead to the same $O(N_{\mathrm {c}}^{-1})$ behavior of the corresponding transition amplitude.

The fact that a single tetraquark satisfies all the existing constraints does not exclude the possibility of the existence of two types of tetraquark, with different internal structures. However, contrary to the exotic case [Eqs. (2.12) and (2.13)], these would not have now preferred decay channels. A particular case may emerge when, for some dynamical reason, tetraquarks do not contribute to diagram (b) of Fig. 10. In that case, one falls back into the situation of the exotic case, where two different tetraquarks, each one having a preferred decay channel, are needed.

We now consider the case of the open-type channel, where the quark flavor *c* appears through two quark fields, rather than a pair of a quark and an antiquark field. The four-point correlation function describing the corresponding meson–meson scattering is

3.12 $$\begin{aligned} \varGamma =\langle j_{ac}^{}j_{bc}^{}j_{bc}^{\dagger }j_{ac}^{\dagger } \rangle . \end{aligned}$$

**Fig. 16:**
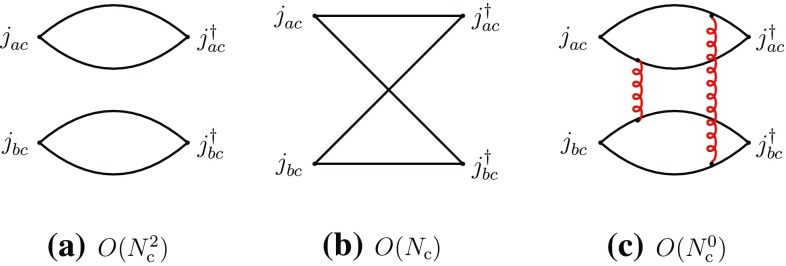
Leading and subleading diagrams of the correlation function (3.12)

Here, the direct and recombination channels are identical, with the common process $M_{ac}^{}M_{bc}^{}\rightarrow M_{ac}^{}M_{bc}^{}$. The corresponding $N_{\mathrm {c}}^{}$-leading and subleading diagrams are represented in Fig. 16.

**Fig. 17:**
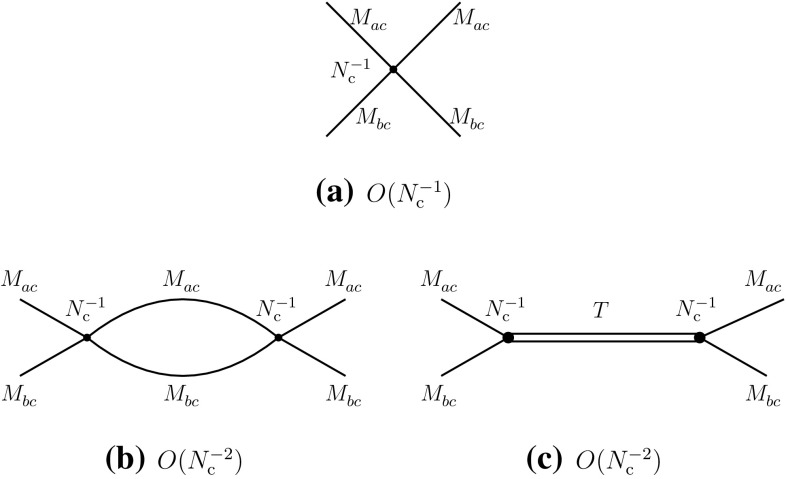
Four-meson vertex (**a**), two-meson intermediate states (**b**) and tetraquark state (**c**) arising from the correlation function (3.12)

Their implication in terms of the four-meson vertex and two-meson and tetraquark intermediate states is shown in Fig. 17.

One also obtains a decay width of the order of $N_{\mathrm {c}}^{-2}$ [Eq. (3.11)].

The case of cryptoexotic channels with two quark flavors can be treated in the same way as for the case with three flavors. The corresponding correlation functions are

3.13 $$\begin{aligned}&\varGamma _{\mathrm {I}}^{\mathrm {dir}}= \langle j_{ac}^{}j_{ca}^{}j_{ca}^{\dagger }j_{ac}^{\dagger }\rangle ,\quad \varGamma _{\mathrm {II}}^{\mathrm {dir}}= \langle j_{aa}^{}j_{cc}^{}j_{cc}^{\dagger }j_{aa}^{\dagger }\rangle ,\nonumber \\&\varGamma ^{\mathrm {rec}}=\langle j_{ac}^{}j_{ca}^{}j_{cc}^{\dagger } j_{aa}^{\dagger }\rangle ,\quad a\ne c. \end{aligned}$$

Most of the relevant diagrams are similar to those found in the three-flavor case. In addition, one finds annihilation-type diagrams involving gluon lines. In particular, the direct channel I of Eq. (3.13) involves, among others, an annihilation diagram with two gluon lines (similar to diagram (c) of Fig. 16, rotated by $\pi /2$), which produces, in meson space, a glueball as an intermediate state in the *s*-channel [the analog of Fig. 4b in the *s*-channel]. The presence of the new diagrams does not change the qualitative results found earlier in the large-$N_{\mathrm {c}}^{}$ limit. Mixings of tetraquarks with glueball states are of subleading order. Therefore, the main conclusions about the tetraquark decay width and two-meson intermediate states remain unchanged.

## Conclusion

The study of compact-tetraquark properties in the large-$N_{\mathrm {c}}^{}$ limit of QCD through the meson–meson scattering amplitudes involves $N_{\mathrm {c}}^{}$-subleading diagrams, at which order also two-meson states occur. It was shown that the latter consistently satisfy the constraints emerging from the $1/N_{\mathrm {c}}^{}$ expansion procedure, thus providing a firm basis for the extraction of the tetraquark properties. Considering many types of quark flavor and various combinations of mesons in the *s*-channel, it was found that, in general, tetraquarks have narrow decay widths, of the order of $N_{\mathrm {c}}^{-2}$, much smaller than those of ordinary mesons. For the particular case of exotic tetraquarks, involving four different quark flavors, two different types of tetraquark are needed to satisfy the consistency constraints, each of them having a preferred decay channel and a quark structure made of the product of two color-singlet bilinears.

In the real world, where $N_{\mathrm {c}}^{}=3$, some of the qualitative aspects found above might be attenuated. Nevertheless, they might still serve as a guidance for quantitative investigations.

## Figures and Tables

**Fig. 18:**
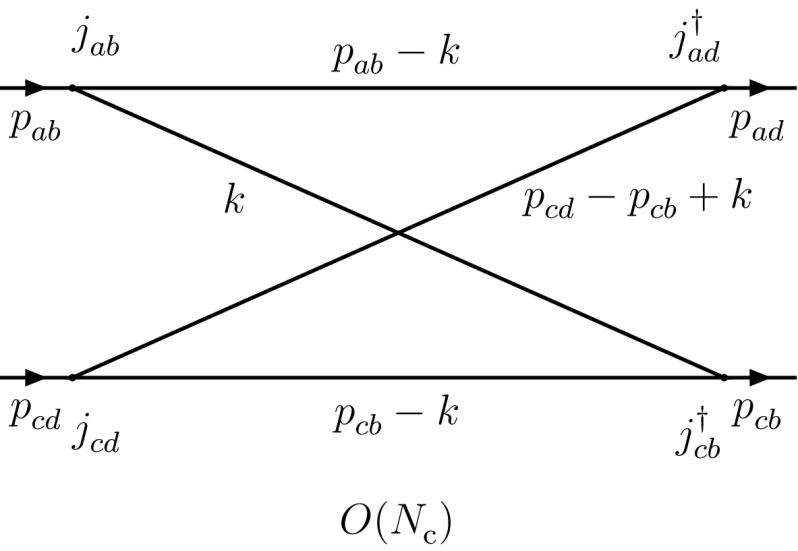
Recombination diagram of Fig. 2a with explicit momentum flow

**Fig. 19:**
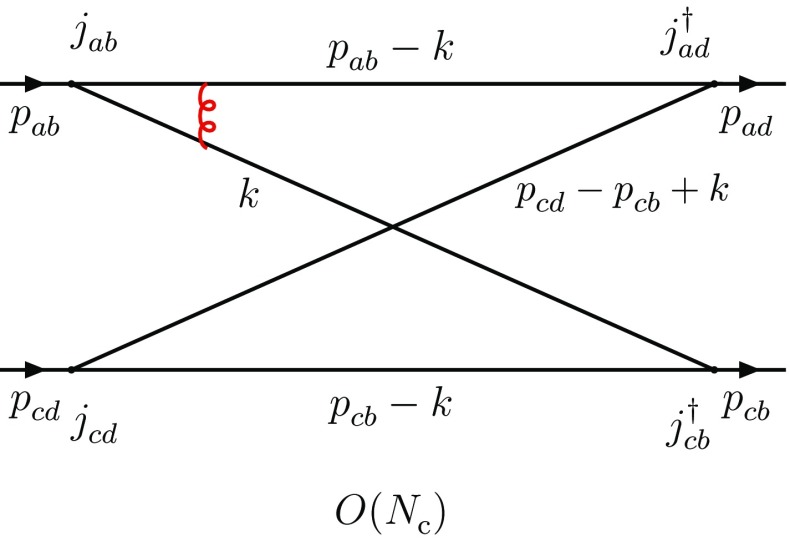
One-gluon exchange between quarks *a* and *b*

**Fig. 20:**
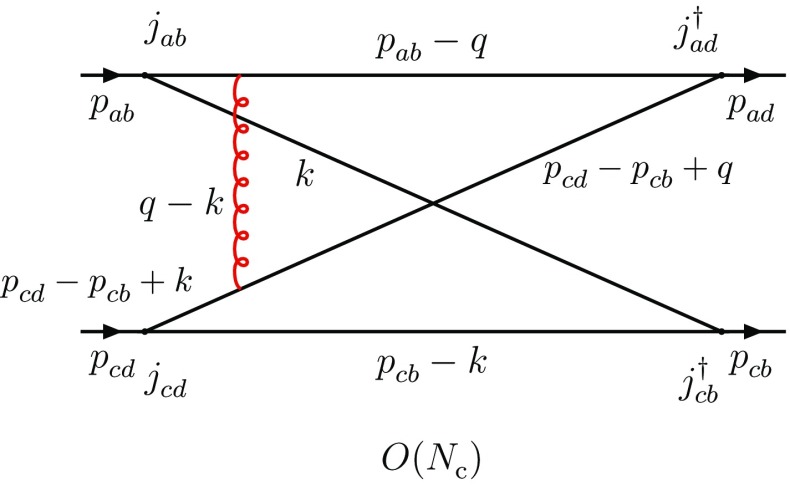
One-gluon exchange between quarks *a* and *d*

**Fig. 21:**
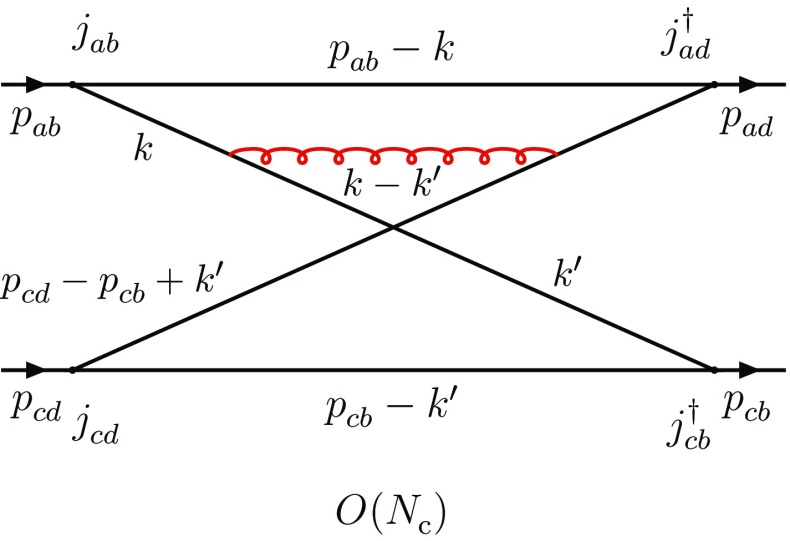
One-gluon exchange between quarks *b* and *d*

**Fig. 22:**
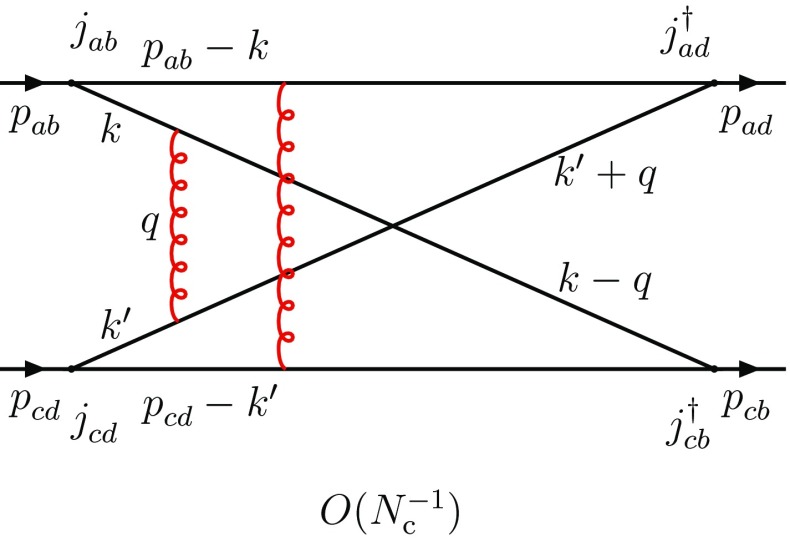
Two-gluon exchanges between the four quarks

**Fig. 23:**
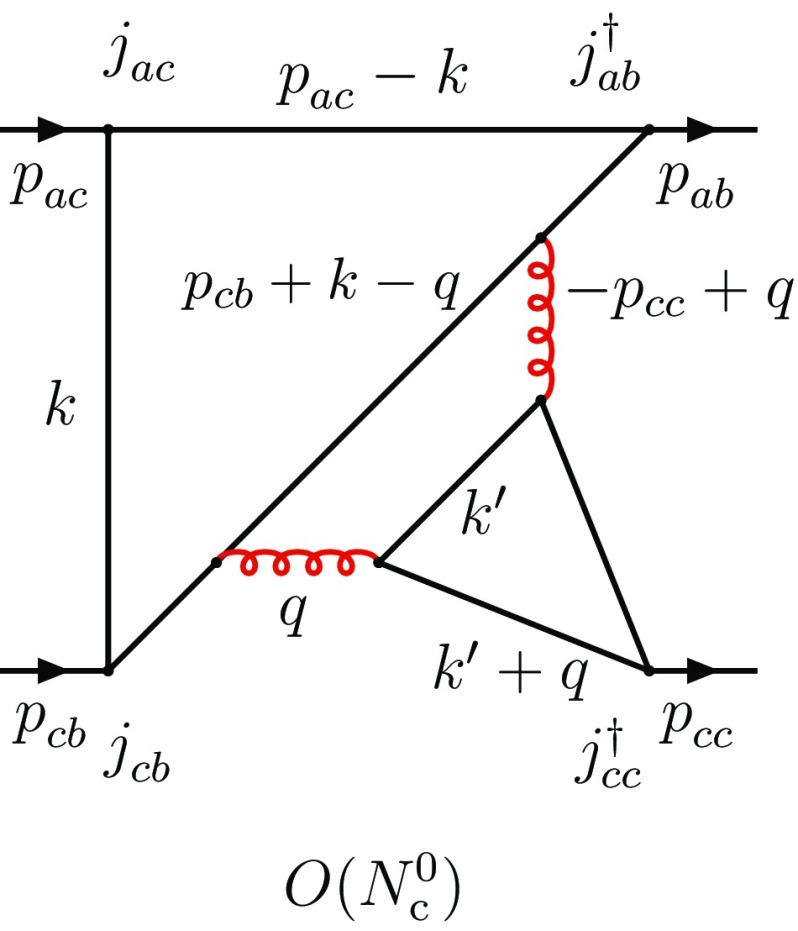
A diagram of the recombination channel of the cryptoexotic case with two-gluon exchanges

**Fig. 24:**
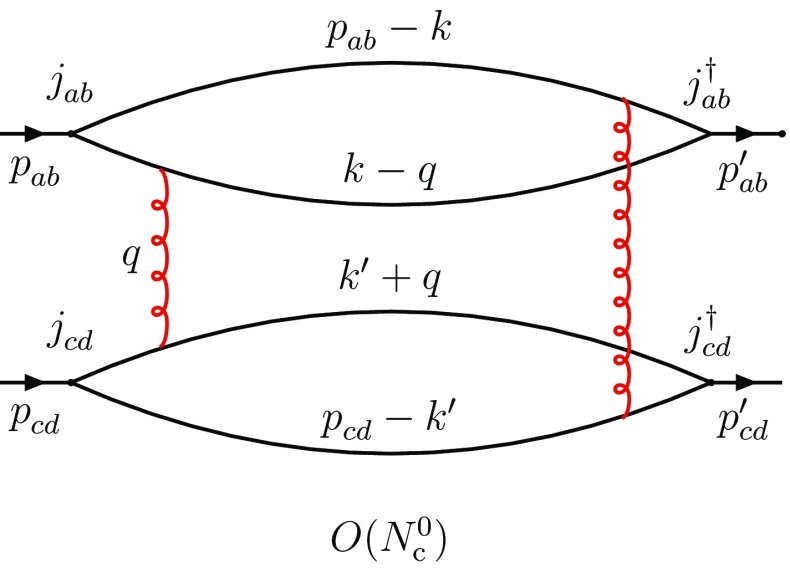
Two-gluon exchanges between the disconnected pieces of the direct channel I [Eq. (2.3) and Fig. 1b]

## Appendix A: Landau equations

For completeness, we present in this appendix several typical cases, where the Landau equations are used for the determination of the location of singularities occurring in Feynman diagrams involved in the large-$N_{\mathrm {c}}^{}$ limit. A generic expression of the latter is

A.1 $$\begin{aligned} I(p)=\int \prod _{\ell =1}^{L}\frac{\mathrm{d}^4q_{\ell }^{}}{(2\pi )^4} \prod _{i=1}^{I}\frac{1}{(k_i^2-m_i^2+i\epsilon )}, \end{aligned}$$

where *p* represents a collection of external momenta and $k_i$ are linear functions of the *p* and of the loop variables *q*.

The Landau equations are [22, 23]

A.2 $$\begin{aligned}&\lambda _i^{}(k_i^2-m_i^2)=0,\quad i=1,\ldots ,I, \end{aligned}$$

A.3 $$\begin{aligned}&\sum _{i=1}^I\lambda _i k_i^{}\cdot \frac{\partial k_i^{}}{\partial q_{\ell }^{}}=0,\quad \ell =1,\ldots ,L, \end{aligned}$$

where the $\lambda$ are parameters (Lagrange multipliers) to be determined.

This system of equations may have independent subsystems, corresponding to the vanishing of a certain number of parameters $\lambda$.

We are mainly interested in the singularities produced by the quark propagators. Gluon propagators may also produce singularities, but since the gluons are massless, and except in annihilation diagrams, these generally start at the same positions as those produced by the quark propagators. We therefore will not consider, in general, gluon propagators in the Landau equations, except in one case, for illustrative purposes; this amounts to taking the corresponding $\lambda$ equal to zero.

However, gluon lines may play a decisive role in the production of quark singularities through the momentum they carry. We can distinguish two types of gluon contribution. For the first category, the gluon lines participate in the renormalization of existing objects, like propagators or vertices. In this case, one could ignore them for the present purpose. For the second category, they participate in the interaction between different clusters of objects and play an active role in the formation of singularities related to intermediate states. Those should be considered, when present.

We shall mainly consider recombination-type diagrams of the exotic channels.

We begin with the recombination diagram of leading order [Figs. 2a and 18].

The external momenta are $p_{ab}^{}$, $p_{cd}^{}$, $p_{ad}^{}$, $p_{cb}^{}$, with the definitions

A.4 $$\begin{aligned}&P=p_{ab}^{}+p_{cd}^{}=p_{ad}^{}+p_{cb}^{},\quad s=P^2,\nonumber \\&t=(p_{ab}^{}-p_{ad}^{})^2,\quad u=(p_{ab}^{}-p_{cb}^{})^2. \end{aligned}$$

The Landau equations are

A.5 $$\begin{aligned}&\lambda _a^{}((p_{ab}^{}-k)^2-m_a^2)=0, \lambda _b^{}(k^2-m_b^2)=0,\nonumber \\&\quad \lambda _c^{}((p_{cb}^{}-k)^2-m_c^2)=0,\nonumber \\&\quad \lambda _d^{}((p_{cd}^{}-p_{cb}^{}+k)^2-m_d^2)=0, \end{aligned}$$

A.6 $$\begin{aligned}&-\,\lambda _a^{}(p_{ab}^{}-k)+\lambda _b^{}k-\lambda _c^{} (p_{cb}^{}-k)\nonumber \\&\quad +\,\lambda _d^{}(p_{cd}^{}-p_{cb}^{}+k)=0. \end{aligned}$$

This system of equations has several independent subsystems. Choosing $\lambda _b^{}=\lambda _d^{}=0$, one obtains $u=(m_a^{}\pm m_c^{})^2$. (Only physical singularities, corresponding to $+$ signs between the masses, are relevant.) Choosing $\lambda _a^{}=\lambda _c^{}=0$, one obtains $t=(m_b^{}\pm m_d^{})^2$. Choosing $\lambda _c^{}=\lambda _d^{}=0$, one obtains $p_{ab}^2=(m_a\pm m_b)^2$, and so forth. The latter type of singularities are present inside the meson propagators. The property that the singularities in *u* and *t* involve only two quark masses is reminiscent of the fact that there are no four-quark singularities. The *u*- and *t*-channel singularities will be saturated by one-meson intermediate states. No singularities in *s* are found.

The general system involving the four $\lambda$ leads to an equation where *u*, *t* and the $p^2$ enter; it comes from the condition of the vanishing of the determinant of a $4\times 4$ matrix. In principle, this equation should give information about the possible existence of anomalous thresholds or unphysical thresholds in the *u*- and *t*-channels, depending on the values taken by the $p^2$. The cases of interest are those where the $p^2$ represent masses squared of the external mesons. The equation considerably simplifies in the equal-mass case. Taking $m_a=m_b=m_c=m_d=m$ and $p_{ab}^2=p_{cd}^2=p_{ad}^2=p_{cb}^2=p^2$, one finds that the singularities occur on the unphysical sheets, at $u=0$ or $t=0$, which are the equal-mass limits of $u=(m_a-m_c)^2$ and $t=(m_b-m_d)^2$, respectively. This shows the general tendancy of the equation of not producing anomalous thresholds on the physical sheet. In any event, no *s*-channel singularities arise.

The next example contains one-gluon exchange between quarks *a* and *b* (Fig. 19).

Here, the loop momenta can be chosen in such a way that the quark propagators, cut by a vertical line on the right of the gluon propagator, have the same momentum dependence as in the preceding example. This shows, as expected, that the gluon does not play any role for the determination of the *s*- or *t*- or *u*-channel singularities. It contributes to the renormalization of the current vertex or to the incoming meson propagator. Cutting the diagram with a vertical line on the left of the gluon propagator does not lead to new singularities.

The third example contains one-gluon exchange between quarks *a* and *d* (Fig. 20).

Taking the vertical cut on the right of the gluon line, one obtains the equations

A.7 $$\begin{aligned}&\lambda _a^{}((p_{ab}^{}-q)^2-m_a^2)=0, \lambda _b^{}(k^2-m_b^2)=0,\nonumber \\&\lambda _c^{}((p_{cb}^{}-k)^2-m_c^2)=0,\nonumber \\&\lambda _d^{}((p_{cd}^{}-p_{cb}^{}+q)^2-m_d^2)=0, \end{aligned}$$

A.8 $$\begin{aligned}&-\,\lambda _a^{}(p_{ab}^{}-q)+\lambda _d^{}(p_{cd}^{}-p_{cb}^{}+q)=0, \nonumber \\&\lambda _b^{}k-\lambda _c^{}(p_{cb}^{}-k)=0. \end{aligned}$$

The system of equations factorizes into two independent subsystems, with solutions $p_{ad}^2=(m_a^{}\pm m_d^{})^2$ and $p_{cb}^2=(m_b^{}\pm m_c^{})^2$.

Taking the vertical cut on the left of the gluon line, one finds the same equations as for Fig. 18, with the same solutions.

For completeness, we also consider the case of a gluon exchanged between quarks *b* and *d*. The case where the gluon line is not cut by the vertical line, corresponds to a figure similar to Fig. 20. With a relabeling of momenta, one obtains the same type of Landau equations, with similar conclusions. For illustration, we consider here the case where the gluon line is cut by the vertical line (Fig. 21).

The Landau equations are

A.9 $$\begin{aligned}&\lambda _a^{}((p_{ab}^{}-k)^2-m_a^2)=0, \lambda _b^{}(k^{\prime 2}-m_b^2)=0,\nonumber \\&\lambda _c^{}((p_{cb}^{}-k')^2-m_c^2)=0, \lambda _g((k-k')^2-m_g^2)=0,\nonumber \\&\lambda _d^{}((p_{cd}^{}-p_{cb}^{}+k')^2-m_d^2)=0, \end{aligned}$$

A.10 $$\begin{aligned}&-\lambda _a^{}(p_{ab}^{}-k)+\lambda _g^{}(k-k')=0, \nonumber \\&-\lambda _g(k-k')+\lambda _b^{}k'-\lambda _c^{}(p_{cb}^{}-k') +\lambda _d(p_{cd}-p_{cb}+k')\nonumber \\&\quad =0, \end{aligned}$$

where we have also attributed a mass $m_g$ to the gluon, in order to have a more explicit control of its effect. The nontrivial solution of these equations corresponds to the case $\lambda _b=\lambda _d=0$, leading to a singularity at $u=(m_a+m_c+m_g)^2$. One verifies that the gluon mass contributes additively to the quark masses in the existing *u*-channel singularity. In the limit where the gluon mass tends to zero, the corresponding threshold tends to the existing two-quark threshold.

The fifth example contains two-gluon exchanges between quarks *a* and *c*, and *b* and *d*, respectively (Fig. 22). The vertical cut is taken between the two gluon lines. The Landau equations are

A.11 $$\begin{aligned}&\lambda _a^{}((p_{ab}^{}-k)^2-m_a^2)=0,\quad \lambda _b^{}((k-q)^2-m_b^2)=0,\nonumber \\&\lambda _c^{}((p_{cd}^{}-k')^2-m_c^2)=0,\quad \lambda _d^{}((k'+q)^2-m_d^2)=0,\nonumber \\ \end{aligned}$$

A.12 $$\begin{aligned}&-\lambda _a^{}(p_{ab}^{}-k)+\lambda _b^{}(k-q)=0,\nonumber \\&-\lambda _c^{}(p_{cd}^{}-k')+\lambda _d^{}(k'+q)=0,\nonumber \\&-\lambda _b^{}(k-q)+\lambda _d^{}(k'+q)=0. \end{aligned}$$

The system of equations can be solved: one finds the physical singularity at $P^2=s=(m_a^{}+m_b^{}+m_c^{}+m_d^{})^2$. (The unphysical singularities, corresponding to changes of sign in front of the masses, exist as well.) The fact that four masses are present means that we have four-quark intermediate states, which then would generate, together with the contributions of other diagrams involving increasing numbers of gluon exchanges, two-meson and eventually tetraquark states.

The reason of the appearance of the *s*-channel singularity is related to the iterative nature of the diagram. Considering, in general, the four-quark Green’s function (eight-point function), the diagrams describing the latter are usually divided into irreducible and reducible types, the latter being obtained by iteration of the former. In the present case, the diagram is one of the first iterations of one-gluon exchange diagrams.

We next consider the recombination diagram (b) of Fig. 10 of the cryptoexotic channels (Fig. 23), involving three different quark flavors.

The vertical cut is taken in the right part of the diagram, crossing the four quark propagators. The Landau equations are

A.13 $$\begin{aligned}&\lambda _a^{}((p_{ac}^{}-k)^2-m_a^2)=0,\quad \lambda _b^{}((p_{cb}^{}+k-q)^2-m_b^2)=0,\nonumber \\&\lambda _c^{}(k^{\prime 2}-m_c^2)=0,\quad \lambda _c^{\prime }((k'+q)^2-m_c^2)=0, \end{aligned}$$

A.14 $$\begin{aligned}&-\lambda _a^{}(p_{ac}^{}-k)+\lambda _b^{}(p_{cb}^{}+k-q)=0, \nonumber \\&-\lambda _b^{}(p_{cb}^{}+k-q)+\lambda _c^{\prime }(k'+q)=0,\nonumber \\&\lambda _c^{}k'+\lambda _c^{\prime }(k'+q)=0. \end{aligned}$$

The above system of equations can be solved, leading to the physical singularity at $P^2=s=(m_a^{}+m_b^{}+2m_c^{})^2$, which is the signal of the presence of four-quark intermediate states.

As a last example, we consider the direct channel I diagram with two-gluon exchanges [Figs. 1b and 24]. The loop momenta can be chosen in such a way that the quark propagators cut by the vertical line between the two gluons have the same momenta as in the case of Fig. 22. Therefore, one finds the same *s*-channel singularities.

## Footnotes

1 Henceforth called for simplicity “four-quark intermediate states”.
